# Effects of sub-chronic, *in vivo* administration of sigma-1 receptor ligands on platelet and aortic arachidonate cascade in streptozotocin-induced diabetic rats

**DOI:** 10.1371/journal.pone.0265854

**Published:** 2022-11-17

**Authors:** Sándor Váczi, Lilla Barna, Krisztián Laczi, Ferenc Tömösi, Gábor Rákhely, Botond Penke, Lívia Fülöp, Ferenc Bogár, Tamás Janáky, Mária A. Deli, Zsófia Mezei

**Affiliations:** 1 Department of Pathophysiology, Albert Szent-Györgyi Medical School, University of Szeged, Szeged, Hungary; 2 Doctoral School of Theoretical Medicine, University of Szeged, Szeged, Hungary; 3 Institute of Biophysics, Biological Research Centre, Szeged, Hungary; 4 Doctoral School of Biology, University of Szeged, Szeged, Hungary; 5 Department of Biotechnology, University of Szeged, Szeged, Hungary; 6 Department of Medical Chemistry, Albert Szent-Györgyi Medical School, University of Szeged, Szeged, Hungary; 7 Department of Physiology, Albert Szent-Györgyi Medical School, University of Szeged, Szeged, Hungary; Max Delbruck Centrum fur Molekulare Medizin Berlin Buch, GERMANY

## Abstract

**Background:**

Diabetes mellitus is a chronic metabolic disorder which induces endothelial dysfunction and platelet activation. Eicosanoids produced from arachidonic acid regulate cellular and vascular functions. Sigma-1 receptors (S1R) are expressed in platelets and endothelial cells and S1R expression is protective in diabetes.

**Objectives:**

Our aim was to examine the influence of sub-chronic, *in vivo* administered S1R ligands PRE-084, *(S)*-L1 (a new compound) and NE-100 on the *ex vivo* arachidonic acid metabolism of platelets and aorta in streptozotocin-induced diabetic rats.

**Methods:**

The serum level of the S1R ligands was detected by LC-MS/MS before the *ex vivo* analysis. Sigma-1 receptor and cyclooxygenase gene expression in platelets were determined by RT-qPCR. The eicosanoid synthesis was examined with a radiolabelled arachidonic acid substrate and ELISA.

**Results:**

One month after the onset of STZ-induced diabetes, in vehicle-treated, diabetic rat platelet TxB_2_ and aortic 6-k-PGF_1α_ production dropped. Sub-chronic *in vivo* treatment of STZ-induced diabetes in rats for one week with PRE-084 enhanced vasoconstrictor and platelet aggregator and reduced vasodilator and anti-aggregator cyclooxygenase product formation. *(S)*-L1 reduced the synthesis of vasodilator and anti-aggregator cyclooxygenase metabolites and promoted the recovery of physiological platelet function in diabetic rats. The S1R antagonist NE-100 produced no significant changes in platelet arachidonic acid metabolism. *(S)*-L1 decreased the synthesis of vasoconstrictor and platelet aggregator cyclooxygenase metabolites, whereas NE-100 increased the quantity of aortic vasodilator and anti-aggregator cyclooxygenase products and promoted the recovery of diabetic endothelial dysfunction in the aorta. The novel S1R ligand, *(S)*-L1 had similar effects on eicosanoid synthesis in platelets as the agonist PRE-084 and in aortas as the antagonist NE-100.

**Conclusions:**

S1R ligands regulate cellular functions and local blood circulation by influencing arachidonic acid metabolism. In diabetes mellitus, the cell-specific effects of S1R ligands have a compensatory role and aid in restoring physiological balance between the platelet and vessel.

## Introduction

Diabetes mellitus is a chronic, progressive disease characterized by abnormal carbohydrate, lipid and protein metabolism. These abnormalities lead to the formation of glycation end products and the accumulation of reactive oxygen species resulting in endothelial dysfunction and platelet activation [1, 2]. The cytokines and eicosanoids synthesized by the impaired endothelium and activated platelets are involved in the development and progression of atherosclerosis [3]. Eicosanoids, which regulate cellular and vascular function, are generated from free arachidonic acid (AA) released from phospholipids (PLs) in the cell membrane by phospholipase A_2_ in the presence of ionized calcium, by cyclooxygenases (COXs), lipoxygenases (LOXs) and specific synthetases [4]. The sigma-1 receptor (S1R), which modulates cellular functions [5–7], is expressed on both endothelial cells [8] and platelets [9]. Beneficial effects of S1R agonists have already been reported in sepsis [10], ischemic-reperfusion injury [11], stroke [12] and cardiovascular disease [13]. The selective S1R agonist PRE-084 (2-(4-morpholino)ethyl-1-phenylcyclohexane-1-carboxylate) [14] can regulate cytokine production in stroke [15], reduce microglial activation in traumatic brain injury, and exert a protective effect in neurogenic inflammation [16] and endothelial barrier damage [17]. NE-100 (N,N-dipropyl-2-[4-methoxy-3- (2-phenylethoxy)-phenyl]-ethylamine monohydrochloride) [18], known as an S1R antagonist, blocks thoracic aortic vasodilation induced by a sigma-1 agonist (SA4503) [13].

In our previous *in vitro* studies we examined the effects of S1R modulation on platelet functions. We demonstrated that the S1R agonist PRE-084 enhanced both eicosanoid synthesis and ADP- or AA-induced aggregation of isolated platelets from healthy rats. In this study, we examined only the acute effect of PRE-084 [9]. In a separate, follow-up study, intraperitoneal sub-chronic *in vivo* PRE-084 treatment reduced the total quantity of *ex vivo/in vitro* COX-mediated AA metabolites in both healthy rat platelets and aorta, although the incubation mixture did not contain S1R ligand. In addition to the S1R agonist PRE-084, the effects of the antagonist NE-100 and a novel ligand, *(S)*-L1 (S-N-Benzyl-6,7-dimethoxy-1,2,3,4-tetrahydro-1-isoquinolineethanamine), on *ex vivo* AA metabolism in healthy rat platelets and aorta were also studied [19].

The ligand *(S)*-L1 was identified by a controlled virtual screening protocol based on its high binding affinity to S1R (K_i_ = 11±3 nM) and moderate sigma-1/sigma-2 receptor selectivity [20] and was chosen to investigate its effects on eicosanoid synthesis in platelets and aorta. The S1R ligands in the screening study all conformed to the early pharmacophore model [21], which consists of the basic amine site required to form an electrostatic bond with Glu172 of the S1R and the hydrophobic groups flanking it. The main difference between agonist and antagonist binding is the presence of an interaction of the ligand with the C-terminal helix of the S1R. The cyclohexane ring of PRE-084 is the only moiety that is able to interact with this helix [22]. The *(S)*-L1 and S1R antagonist NE-100 have very similar binding positions [19, 20].

*In vivo* sub-chronic treatment of healthy rats with *(S)*-L1 resulted in a similar but greater change in platelet AA metabolism than PRE-084, while NE-100 ligand induced an opposite alteration in platelet eicosanoid synthesis to both the ligands PRE-084 and *(S)*-L1 [19].

It is well established in the literature that S1R gene expression reduces the production of inflammatory cytokines [10], reactive oxygen species and advanced glycation end products [2, 3] as well as abnormal protein accumulation in diabetes [23] and protects against the development of diabetic complications [24]. Based on these observations and our previous studies, the aim of the present study was to investigate the effects of sub-chronic intraperitoneal-administered S1R ligands PRE-084, *(S)*-L1 and NE-100 on platelet and aortic eicosanoid synthesis in streptozotocin (STZ)-induced diabetic rats.

We hypothesized that (1) S1R gene (*Sigmar1*) expression increases in diabetic rat platelets *ex vivo* suggesting a compensatory role of S1R in diabetic metabolic alterations; (2) *In vivo* activation of platelet and endothelial dysfunction can be confirmed even after one month of disease and detected by *ex vivo* studies of platelets and aorta in STZ-induced diabetic rats; (3) The S1R agonist PRE-084 and the antagonist NE-100 have opposite effects on the levels of *Sigmar1* and COX (prostaglandin G/H synthase; *Ptgs*) gene mRNA in diabetic rat platelets, whereas the novel S1R ligand *(S)*-L1 acts as an antagonist under the present conditions due to its similar structure to NE-100; (4) S1R ligands administered sub-chronically *in vivo* are able to modulate *ex vivo* platelet and aortic AA metabolism, despite the absence of ligand in the incubation mixture; and (5) Abnormal AA metabolism in platelets and aorta of diabetic animals is modified by S1R ligands in such a way that the physiological balance between them is restored.

To confirm these hypotheses, we measured the serum level of the S1R ligands at the end of sub-chronic treatment and the mRNA levels of *Sigmar1* and *Ptgs* genes in platelets and examined the eicosanoid synthesis in platelets and aorta *ex vivo* in diabetic rats treated with S1R ligands and compared the results to a vehicle-treated, healthy control group.

## Materials and methods

### Animals

The animal experiments were performed based on the protocol approved by the Ethics Committee for the Protection of Animals in Research at the University of Szeged, Hungary (Permit No. X./238/2019.). All the experiments were carried out in accordance with the Guide for the Care and Use of Laboratory Animals published by the U.S. National Institutes of Health. Male Wistar *(Rattus norvegicus)* rats were used in this study. Inclusion in the trial was based on good health, normal serum glucose level, age-appropriate food and fluid intake, and body weight. All the animals fulfilled these criteria, so exclusion from the study was not necessary [25]. After weaning and simple randomization [26] to minimize the effects of subjective bias [27], three rats were placed in a transparent-walled cage. These housing conditions were used to reduce the isolation and environmental discomfort caused by diabetes symptoms in rats. As required by guidelines and the ethics permit, we placed enrichment devices (e.g. cylinders and cubes) in the rat cages. However, it is not possible to reduce the mild pain caused by the procedures with medication, as this would affect our results. All the animals were maintained in a room in 12-h dark/12-h light cycles at constant temperature (23±1°C) with free access to standard laboratory food (SAFE® 132, SAFE, Augy, France) and water *ad libitum*. Sample size calculation was performed for the total quantity of cyclooxygenase metabolites throughout the study. The estimation was based on the one-way analysis of variance (ANOVA) hypothesis test. We supposed the effect size to be at least 0.6, with statistical power 1–β = 0.8, the calculation resulted in a sample size of nine per group with 36 animals in total. Sample size calculation was performed with G*Power version 3.9.1.7 software.

### Diabetic animal model

After a week of adaptation to the environment and handling, diabetes mellitus was induced in eleven-week-old, male rats (n = 36 animals) with a single intraperitoneal (i.p.) injection of 65 mg/kg body weight STZ (Sigma, St. Louis, MO, USA) dissolved in a freshly prepared 50 mM citrate buffer just before use. After STZ injection, the animals’ drinking water was replaced with 10% (w/v) sucrose solution for 24 h [28]. The rats were considered diabetic if the fasting (12 h) peripheral blood glucose concentration was higher than 20 mM 72 h after the injection of STZ (initial serum glucose level). Serum glucose level was monitored by D-count equipment and Ideal test strips (77 Elektronika Ltd., Budapest, Hungary). All the animals that received STZ developed diabetes with several fold blood glucose elevation. We reduced the discomfort caused by the animals’ diabetes symptoms by changing the litter daily. A qualified person checked the health and well-being of the animals daily to decide on the need to terminate the animals. The criteria for termination were cessation of food or fluid intake and loss of body weight or the onset of an unexpected complication. No such event occurred in this series of experiments. No mortality was observed, and therefore no animals had to be excluded.

### Experimental animal groups and study design

Four weeks later, the diabetic animals were randomly divided into four groups (n = 9 animal/group); one of these groups was treated with S1R ligand vehicle (isotonic saline), while the other three groups were treated with the different S1R ligands. Therefore, the generated subgroups were as follows: (1) the control/vehicle-treated, (2) the PRE-084-treated, (3) the *(S)*-L1-treated, and (4) the NE-100-treated groups, each consisting of nine rats.

In order to ensure absolute control, a healthy group of nine animals treated with S1R ligand vehicle was established in parallel with these four diabetic groups. Detailed information can be found in the S1 Appendix.

We monitored basal, initial (72 h after STZ administration) and terminal (immediately before *ex vivo/in vitro*) fasting blood glucose levels and body weight in all the animal groups. Detailed information can be found in the S2 Appendix. Serum glucose was determined from blood obtained from rat tails using a D-count blood glucose meter and an Ideal test strip. Before the *ex vivo/in vitro* eicosanoid synthesis studies (terminal phase), platelet counts and serum levels of S1R ligands were determined in all the rats from blood taken from the abdominal aorta. After serum S1R ligand determination, the remaining plasma of the animals in the same group was randomly divided into three pools, and serum total cholesterol (colorimetric methods), alanine aminotransferase (ALT, IFCC methods/pyridoxal phosphate activation) and urea (UV kinetic reaction) by COBAS 8000 instrument (Roche, Hungary) were determined from these pooled samples.

Determining *Sigmar1* and *Ptgs* mRNA levels required 10^10^ platelets per sample. After testing laboratory parameters and AA metabolism, the remaining blood of one animal no longer contained a sufficient number of platelets for this measurement. To guarantee a suitable platelet count for these tests, we used pooled samples from three rats, which were selected randomly from the nine-animal group, thus yielding a total of three samples. Detailed information on the sampling procedure can be found in the S3 Appendix.

### *In vivo* treatment of rats with S1R ligands

#### Treatment protocols

Four weeks after the STZ injection, the animals were treated with the S1R ligands. All S1R ligand-treated, diabetic animals received i.p. 3 mg/kg body weight of S1R ligand (PRE-084 or *(S)*-L1 or NE-100) dissolved in 0.9% sodium chloride. All the vehicle-treated, diabetic animals received i.p. 0.9% sodium chloride solution. Treatment was carried out once a day for a week in all cases. The number of animals/group and the dose of S1R ligands were determined based on a previous publication describing the effects of a S1R ligand applied i.p. in rats [12].

#### Determination of serum S1R ligands levels

*Reagents and chemicals*. All reagents and chemicals were of analytical or LC–MS grade. Acetonitrile (ACN), methanol (MeOH) and water were obtained from VWR Chemicals (Monroeville, PA, USA). Formic acid (FA) and hydrochloric acid were purchased from Fisher Scientific (Portsmouth, NH, USA). Stock solutions were dissolved in water individually at a final concentration of 1 mg/mL. All standard stock solutions were prepared on ice, divided into 100 μL aliquots and stored at −80°C until further use [19]. S1R ligands: the PRE-084 (2-(4-morpholino)ethyl-1-phenylcyclohexane-1-carboxylate, MedChem Express (USA); the *(S)*-L1 (S-N-Benzyl-6,7-dimethoxy-1,2,3,4-tetrahydro-1-isoquinolineethanamine), a novel high-affinity S1R ligand screened in silico from the in-house compound library at the Institute of Pharmaceutical Chemistry, University of Szeged (Hungary) [20]; the NE-100 (N,N-dipropyl-2-[4-methoxy-3- (2-phenylethoxy)-phenyl]-ethylamine monohydrochloride, Tocris Bioscience (Bristol, UK). 0758–003 is a homologue of *(S)*-L1 used as the internal standard in LC-MS.

*Preparation of plasma samples for the liquid chromatography-mass spectrometry/mass spectrometry (LC-MS/MS)*. According to the results of our preliminary recovery studies [19], 10 and 330 μL ACN (in the assay of *(S)*-L1 and NE-100, respectively) or 330 μL MeOH (in the assay of PRE-084) were added to a 100 μL rat plasma sample. The organic solvents contained 10 μL internal standard (N-benzyl-2-[6,7-dimethoxy-1,2,3,4-tetrahydroisoquinolin-1-yl]propan-1-amine) solution with a concentration of 120 nM (in the assay of *(S)*-L1) or 48 nM (in the assay of PRE-084 and NE-100). The mixture was spun for 60 s and allowed to rest for 30 min at −20°C to support protein precipitation. The supernatant was obtained via centrifugation of the mixture for 15 min at 15,000×g at 4°C. The supernatant was transferred to a new tube. After concentration under vacuum (Savant SC 110 A Speed Vac Plus, Savant, USA), the samples were reconstituted in 100 μL starting eluent, vortexed and centrifuged. Finally, 2 μL was injected into the LC-MS/MS system for analysis [19].

*The calibration curves for (S)-L1*, *PRE-084 and NE-100*. The rat plasma calibration standards of *(S)*-L1 (7.81–250 nM), PRE-084 and NE-100 (3.91–125 nM) were prepared by adding the working standard solutions (instead of 10 μL 0.01 M HCL) into a pool of drug-free rat plasma [19]. The sample preparation procedure described above was followed.

*Analysis of plasma samples by the LC-MS/MS*. A quantitative analysis of *(S)*-L1, PRE-084 and NE-100 was conducted after chromatographic separation using tandem mass spectrometry. An ACQUITY I-Class UPLC™ liquid chromatography system (Waters, Manchester, UK) comprising Binary Solvent Manager, Sample Manager-FL and Column Manager connected to a Q Exactive™ Plus Hybrid Quadrupole-Orbitrap Mass Spectrometer (Thermo Fisher Scientific, San Jose, CA, USA) equipped with a heated electrospray ion source (HESI-II) was used for the analysis. Gradient chromatographic separation was performed at room temperature on a Kinetex EVO C18 column (Phenomenex; 100 Å, 50 mm×2.1 mm, particle size 2.6 μm) protected by a C18 guard column (Phenomenex, Torrance, CA, USA) using 0.1% (v/v) aqueous FA as solvent A and ACN containing 0.1% (v/v) FA as solvent B.

The calibration curve was shown to be linear over the concentration range discussed above. The parallel reaction monitoring (PRM) data acquisition mode was selected with a quantitative mass spectrometric analysis of *(S)*-L1, PRE-084 and NE-100 using MS/MS. The optimal fragmentation conditions and collision energies of each analyte were identified to achieve the best precursor/product transition for quantitation and maximum sensitivity. The mass spectrometer was used in positive mode with the following parameters of the HESI-II source: spray voltage at 3.5 kV, capillary temperature at 253°C, aux gas heater temperature at 406°C, sheath gas flow rate at 46 L/h, aux gas flow rate at 11 L/h, sweep gas flow rate at 2 L/h and S-lens radio frequency level at 50.0 (source auto-defaults). PRM mode was used for quantification by monitoring the transitions of the quantifier and qualifier ions. A divert valve placed after the analytical column was programmed to switch flow onto the mass spectrometer only when analytes of interest were eluted from the column (1.5–3.0 min) to prevent excessive contamination of the ion source and ion optics. The washing procedures of the auto sampler before and after injecting samples were programmed to avoid carryover of analytes. The UHPLC system was checked using MassLynx 4.1 SCN 901 (Waters). The mass spectrometer, data acquisition and data processing were checked with Xcalibur™ 4.1 (Thermo Fisher Scientific) [19].

### Separation of platelets

Blood was drawn from the rats 20 hours after the last injection of S1R ligands had been administered. Under anesthesia (Euthasol®/pentobarbital-Na/ 30 mg/kg body weight i.p.), blood was drawn from the abdominal aorta of rats with a thick needle and diluted (1:2) with phosphate buffer (pH 7.4) containing ethylenediaminetetraacetic acid (EDTA, 5.8 mM) and glucose (5.55 mM). The platelets were separated by differential centrifugation [29, 30]. The platelet-rich plasma was collected after the whole blood had been centrifuged at 200 g for 10 min at room temperature. The platelets were sedimented from the supernatant by centrifugation at 2000 g for 10 min. The pellet was contaminated with red blood cells; the erythrocytes were therefore lysed with hypoosmotic ammonium chloride (0.83%, 9 parts) containing EDTA (0.02%, 1 part) over 15 min. The platelets were then washed twice with phosphate buffer pH 7.4 containing 5.8 mM EDTA and 5.55 mM glucose and centrifuged at 2000 g for 10 min at room temperature to remove the ammonium chloride and the erythrocyte residue/debris. The absolute platelet count was determined before the second centrifugation. After the last centrifugation, the platelets were re-suspended (2.5 x 10^8^ platelets/mL) in serum-free Medium 199 tissue culture (Sigma, St. Louis, MO, USA) [19].

### Determination of *Sigmar1*, *Ptgs1* and *Ptgs2* gene expression with RT-qPCR

After simple randomization [26] blood samples from the nine rats in the treatment groups were allocated to three subgroups. The equal quantity of blood samples (~7 mL) was pooled, thus resulting in three biological samples (of ~21 mL)/treatment group. Platelets were isolated separately from the pooled blood samples (each representing three animals) and used for RNA isolation. The three biological samples from each treatment group represent a total of nine rats. Rat platelet samples were homogenized in TRI Reagent (Sigma-Aldrich, USA), and then RNA from each sample was transcribed to complementary DNA using a High Capacity cDNA Reverse Transcription Kit (Applied Biosystems, USA) according to the manufacturer’s protocol based on random priming. The RT-qPCR was performed with two technical parallels from one biological sample (total of four reactions/group) using TaqMan Gene Expression Master Mix (Life Technologies, USA) in a Bio-Rad C1000 Touch Thermal Cycler (Bio-Rad Laboratories, USA). Inventoried TaqMan Gene Expression Assays (Life Technologies, USA) were the following: *Sigmar1*—Rn00578590_m1; *Ptgs1*—Rn00566881_m1; *Ptgs2*—Rn01483828_m1; glyceraldehyde 3-phosphate dehydrogenase (Gapdh)—Rn01749022_g1. After heat activation at 95°C for 3 min, the cycling conditions were the following: denaturation for 30 s at 95°C and amplification for 30 s at 60°C (40 cycles). qPCR data were analyzed with Bio-Rad CFX Maestro software (Bio-Rad Laboratories, USA). In all the samples, the transcript level of a gene was normalized to an endogenous control gene (Gapdh, ΔCt = Ct gene–Ct Gapdh). Then ΔΔCt was calculated in comparison with the relative expression of the target genes in the vehicle-treated control groups. Fold changes were calculated using the 2^−ΔΔCt^ formula [19].

### Collection of aorta samples

Under anesthesia (Euthasol®/pentobarbital-Na/ 30 mg/kg body weight i.p.), the abdominal aorta of the rat was excised from the branch of the iliac artery to the diaphragm after blood collection. At 4°C temperature, the connective tissue was removed from the aorta, which was sliced into 1–2 mm thick rings with care so as not to damage the endothelium [19, 31].

### Examination of arachidonic acid metabolism

#### Analysis of eicosanoid synthesis by isotope-labelled arachidonic acid

An *ex vivo* examination of the eicosanoid synthesis of platelets was carried out in Medium 199, which consists of mineral salts, nucleotides, amino acids, vitamins, carbohydrates and Ca^2+^ in the same quantity as the extracellular concentration but without fibrinogen or other plasma proteins. Rat platelets (2.5×10^8^ platelets/mL in each sample) from the four diabetic treatment groups were pre-incubated at 37°C for 3 min, while the rat abdominal aortic rings (15 mg wet mass/mL) were pre-incubated for 10 min in Medium 199 (Merck). The enzyme reaction examined started after pipetting the tracer substrate 1-^14^C-arachidonic acid (3.7 kBq, 0.172 nM in each sample; American Radiolabeled Chemicals, Inc., St. Louis, MO, USA) into the incubation mixture. The enzyme reaction was stopped by bringing the pH to 3 with formic acid after 13 minutes of incubation in the case of the platelet samples and after 30 minutes of incubation in the case of the aortic samples. All of the samples were extracted with ethyl acetate, and the organic phases were evaporated. The residues were reconstituted in ethyl acetate and quantitatively applied to silica gel G thin-layer plates (Kieselgel G 60/DC-Fertigplatten, Merck, Art. 5721). Although the eluent front is straight and the wind effect is insignificant when using positive-pressure thin-layer chromatography, the order of loading the samples onto the thin-layer plate was changed for each new series of tests to prevent these technical errors. The plates were developed to a distance of 16 cm in the organic phase of ethyl acetate:acetic acid:2,2,4-trimethylpentane:water (110:20:30:100) using overpressure thin-layer chromatography (Chrompress 25, Labor MIM, Hungary) [31–34].

A semi-quantitative analysis of labelled AA metabolites was performed with a BIOSCAN AR-2000 Imaging Scanner (Eckert & Ziegler Radiopharma, Berlin, Germany) using Win-Scan 2D Imaging Software. The radiolabelled products of AA were identified with unlabelled authentic standards [29]. Assuming that the exogenously administered labelled AA as a tracer is converted in the same way as the endogenous source, our method allows us to measure the relative quantity of various prostanoids [19, 31].

#### Analysis of eicosanoid synthesis by ELISA

The course of this procedure is the same as that of the examination of radioactive AA metabolism (see above), but, in this case, the radioactive-labelled substrate is not added to the incubation mixture. After incubation, the incubation mixture, which contained either platelets or aortic rings, was placed at –80°C and stored at this temperature until the assay was performed. Before performing the ELISA test, the samples were lyophilized and the lyophilizates were re-suspended in the 310 μL 0.9% NaCl solution as the original incubation medium. The prepared samples were centrifuged at 3000 rpm, for 10 min to remove cell debris. Subsequently, the quantity of COX-1 (sensitivity: 0.285 ng/mL; assay range: 0.3–60 ng/mL) and COX-2 (sensitivity: 0.446 ng/mL; assay range: 0.5–150 ng/mL) was determined in the diabetic rat platelets and aorta using ELISA kits (Shanghai Sunred Biological Technology Co. Ltd, PRC) in compliance with the protocols attached. The optical absorbance of the samples was measured at 450 nm using a STAT FAX 2100 ELISA plate reader (Awareness Technology, Inc., Palm City, FL, USA). Based on the calibration curves, the respective concentrations of the enzymes examined were determined with the SPSS 22.0 software [9].

### Statistical analysis

The normality of the results was checked with Q-Q plots and Kolmogorov–Smirnov test. As the variances were different with Levene’s test, Welch’s analysis of variance (ANOVA) was performed followed by Dunnett’s T3 post hoc test. The actual significance level is noted for each table or figure. The results are expressed as means±SD. The statistical analysis was performed by SPSS version 22.0 (IBM Corp. Released 2013. IBM SPSS Statistics for Windows, Version 22.0. Armonk, NY, USA) and GraphPad Prism (GraphPad Software, San Diego, CA, USA).

## Results

All the results from the healthy, vehicle-treated rats, obtained in parallel with the diabetic animals, can be found in Váczi et al. (doi: 10.1016/j.ejphar.2022.174983) [19]. All data used in this manuscript have been uploaded to a data repository and made publicly available at link https://osf.io/5wgdb/files/osfstorage.

### Physical and laboratory parameters of the animals

#### STZ-induced diabetic model

As checked in the initial and the terminal phase, all the STZ-treated animals were hyperglycaemic. The fasting blood glucose levels were ~5 times higher than the basal glucose levels (Table 1) or those of the vehicle treated healthy rats tested in parallel with the present experiment [19]. In addition to hyperglycaemia, the development of diabetes was confirmed by the fact that the body weight gain of all the STZ-treated animals was significantly lower in the terminal phase as compared to that of the vehicle-treated, healthy rats. There were no significant differences between terminal blood glucose levels and body weight in diabetic animals receiving different treatments (vehicle, PRE-084, *(S)*-L1 and NE-100). The platelet counts were also in the same range, except for the NE-100 treatment group, in which an elevated terminal platelet count was measured (Table 1).

**Table 1:** Body weight, fasting serum glucose levels and platelet number of diabetic rats.

Rats	Body weight (g)			Serum glucose (mM)			Platelet (10 ^9^ /L)
	*Basal*	*Initial*	*Terminal*	*Basal*	*Initial*	*Terminal*	*Terminal*
**Vehicle**	314*±*25	319*±*23	340*±*33	6.5*±*0.3	29*±*2.9^ooo^	32*±*2.7^ooo^	522*±*79
**PRE-084**	317*±*36	322*±*36	347*±*42	6.4*±*0.3	29*±*3.1^ooo^	33*±*2.3^ooo^	591*±*132
***(S)*-L1**	326*±*13	331*±*13	369*±*27	6.5*±*0.4	28*±*4.1^ooo^	32*±*3.8^ooo^	507*±*86
**NE-100**	315*±*15	320*±*16	333*±*18	6.5*±*0.3	30*±*2.4^ooo^	32*±*2.4^ooo^	635*±*67^#^

Data is shown as mean±SD; n = 9 animals per group; Welch’s ANOVA, Dunnett’s T3 test, ^ooo^p<0.01 initial or terminal serum glucose levels were compared to the basal serum glucose levels.

^**#**^p<0.05 terminal platelet number of NE-100-treated diabetic rat was compared to the vehicle-treated, diabetic rats.

**Fig 1:**
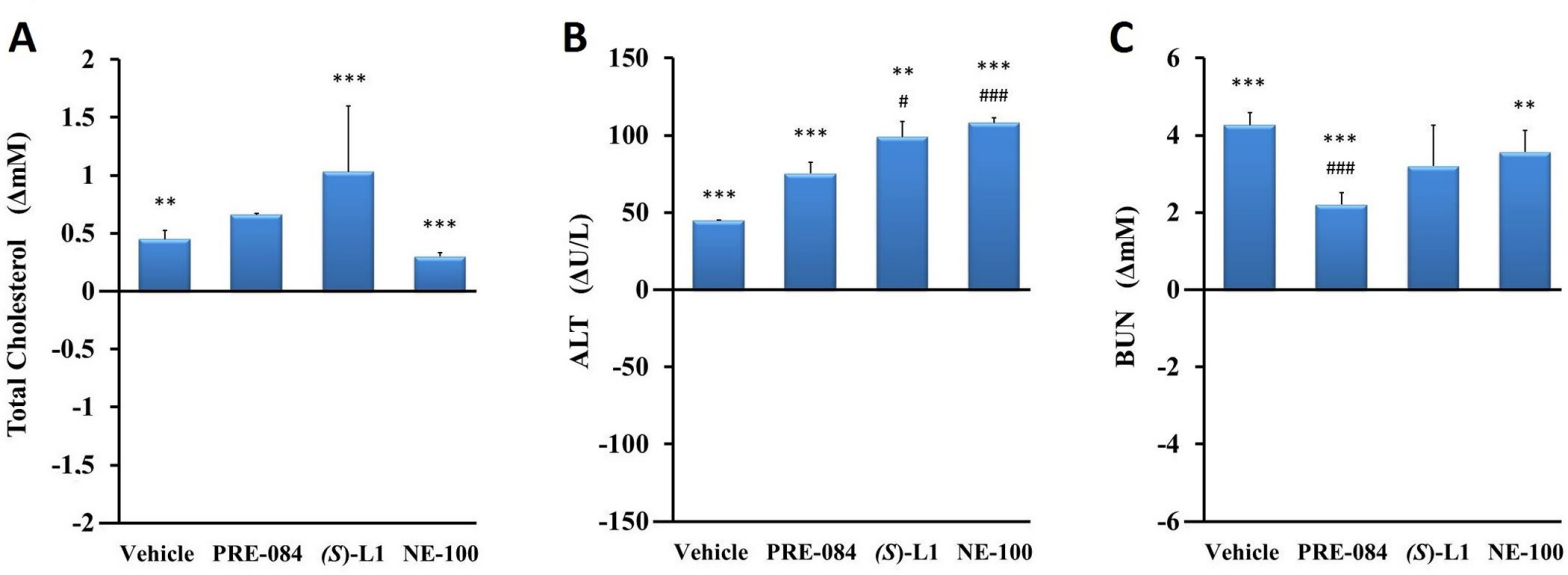
Laboratory parameters of rats at the end (terminal phase) of the *in vivo* study. Plasma total cholesterol (A), alanine aminotransferase (ALT) (B), and blood urea nitrogen (BUN) (C) levels in diabetic vehicle, PRE-084, *(S)*-L1 or NE-100-treated rats are shown as compared to the vehicle-treated, healthy animals. The zero line in the diagram shows the mean of the plasma concentrations in the vehicle-treated, healthy rats, while the columns in the figure represent the delta mean±SD values for the diabetic treatment groups; n = 3 samples pooled from nine rats per group; Welch’s ANOVA, Dunnett’s T3 test, **p<0.03, ***p<0.01 compared to the vehicle-treated, healthy rat group; ^#^p<0.05, ^###^p<0.01 compared to the vehicle-treated, diabetic rat group.

At the end of the *in vivo* study (terminal phase), the levels of total cholesterol (Chol), alanine aminotransferase (ALT) and blood urea nitrogen (BUN) were determined from the pooled plasma samples (Fig 1).

The serum Chol, ALT and BUN levels in the STZ-induced diabetic animals treated with vehicle were higher (Chol by 21%; ALT by 120%; BUN by 63%) than those of the healthy rats studied in parallel with the present experiment. PRE-084 and NE-100 significantly elevated serum Chol and BUN in the diabetic rats compared to the vehicle-treated, healthy animals. In the diabetic rats, all three S1R ligands further enhanced the STZ-induced increase in plasma ALT levels compared to the vehicle-treated, healthy animals. None of the S1R ligands tested significantly altered the rise in serum Chol levels induced by STZ treatment. *(S)*-L1 and NE-100 further enhanced the growth in ALT levels observed in the vehicle-treated, diabetic rats. The smallest growth in ALT was detected when PRE-084 was used. An STZ-induced rise in BUN levels was only attenuated by PRE-084 (Fig 1).

### Concentration of S1R ligands in rat plasma

In our preliminary experiments, we demonstrated that i.p. administered S1R ligands appear in the circulation within one hour, but their plasma levels are at the limit of detection after 20 hours [19]. Plasma concentrations of S1R ligands were determined in all diabetic rats 20 hours after the last ligand injection, immediately before the *ex vivo* studies. In the diabetic rats, the plasma concentration of PRE-084 was below the quantification limit, that of *(S)*-L1 was 10.99±1.06 mM, and that of NE-100 was 5.31±3.14 mM (data mean±SD; n = 9 rats per group). PRE-084 was eliminated from the circulation the fastest of all the Sigma-1 ligands we tested, while *(S)*-L1 was eliminated the slowest [19].

### Effects of S1R ligands on the level of *Sigmar1* and *Ptgs1* transcripts in the diabetic rat platelets

**Fig 2:**
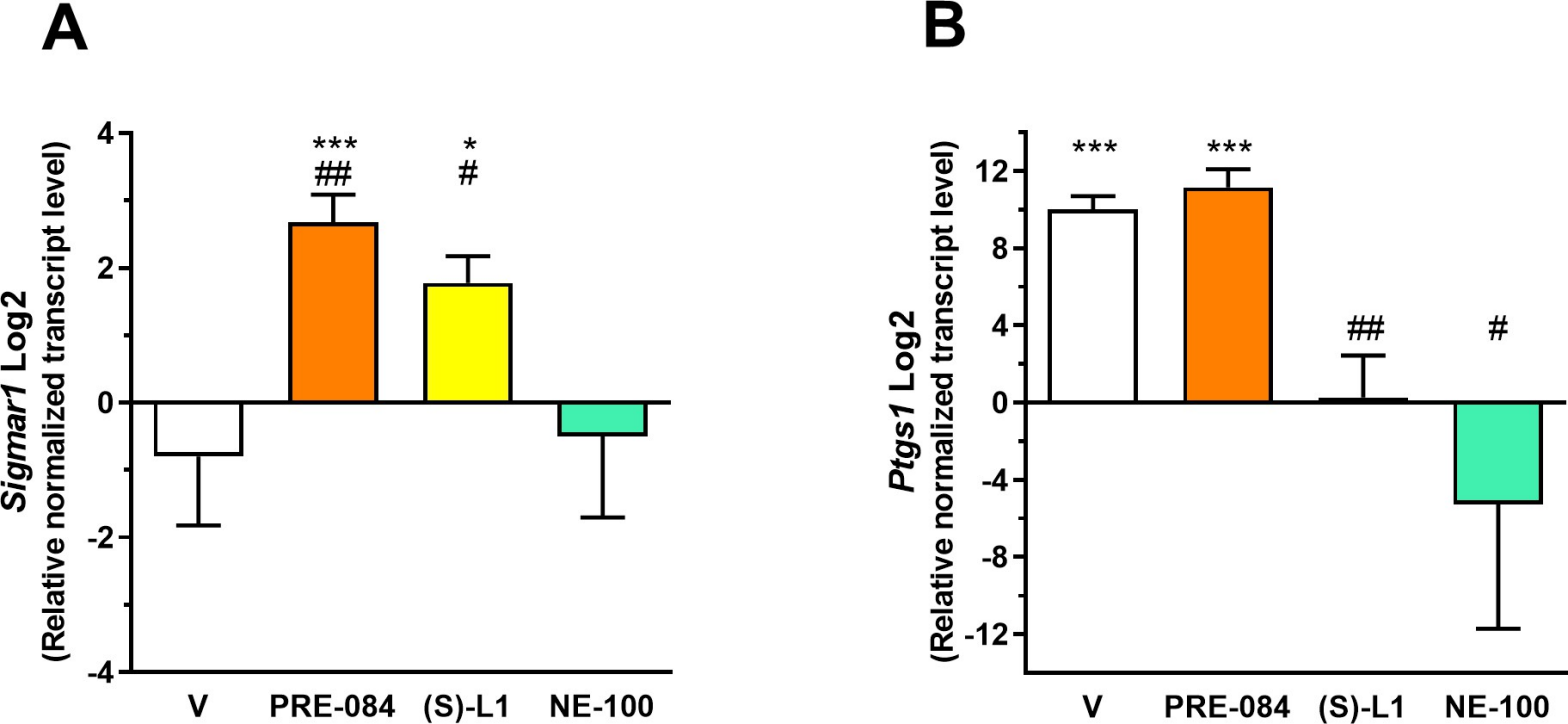
Relative transcript levels of *Sigmar1 and COX-1 (Ptgs1)* genes in platelet samples from diabetic rats treated with S1R ligands normalized to the healthy control group. The diabetic rats were treated with PRE-084, *(S)*-L1, NE-100 or saline solution (V, vehicle) for seven days. The transcript levels of *Sigmar1* (A) and *Ptgs1* (B) genes in the rat platelet samples were first normalized to their own endogenous control mRNA levels (*Gapdh*) and then to the similarly normalized mRNA levels in the vehicle-treated, healthy animal group. Fold changes were calculated using the 2^−ΔΔCt^ formula. Mean±SD, n = 4 representing nine rats per each group, Welch’s ANOVA, Dunnett’s T3 test, *p<0.05, ***p<0.001 compared to the vehicle-treated, healthy group; ^#^p<0.05, ^##^p<0.01 compared to the vehicle-treated, diabetic group.

The mRNA levels of *Sigmar1* were detected in rat platelet samples using RT-qPCR (Fig 2).

A low concentration level of the *Sigmar1* mRNA was detected in the rat platelets from the vehicle-treated, diabetic animals (Fig 2A), similarly to the platelet samples from the healthy rats as published in our previous study [19]. Daily administration of PRE-084 and *(S)*-L1 for one week to diabetic rats elevated *Sigmar1* transcript levels as compared to samples from the vehicle-treated, diabetic or healthy rats (Fig 2A) [19] rats. The *Sigmar1* mRNA level was lower in the NE-100-treated diabetic group compared to those in the PRE-084 and *(S)*-L1-treated samples but did not differ significantly from the vehicle-treated, diabetic (Fig 2A). or healthy groups.

The level of *Ptgs1* transcript was significantly higher in the platelets of the vehicle-treated, diabetic rats compared to those of the vehicle-treated, healthy animals (Fig 2B). In the diabetic rats, PRE-084 treatment did not change *Ptgs1* mRNA levels as compared to samples from the vehicle-treated, diabetic animals. The *Ptgs1* mRNA level was lower in the *(S)*-L1 group than in the vehicle or PRE-084-treated diabetic rat group, but did not differ significantly from the vehicle-treated, healthy group (Fig 2B). NE-100 treatment in diabetic rats significantly decreased the platelet *Ptgs1* level as compared to the vehicle-treated, diabetic group (Fig 2B). Interestingly, the effect of *(S)*-L1 was different in the case of *Sigmar1* (Fig 2A) and *Ptgs1* (Fig 2B) mRNA levels: an effect similar to the S1R agonist PRE-084 was observed for *Sigmar1* mRNA level (Fig 2A), while a trend similar to the S1R antagonist NE-100 was seen for the *Ptgs1* mRNA concentration in the rat platelets (Fig 2B). *Ptgs2* mRNA was not detected in the platelet samples by RT-qPCR using 40 cycles in the diabetic or healthy rats.

### Analysis of *ex vivo* AA metabolism in diabetic rats

#### Effects of S1R ligands on the *ex vivo* eicosanoid synthesis of platelets

**Fig 3:**
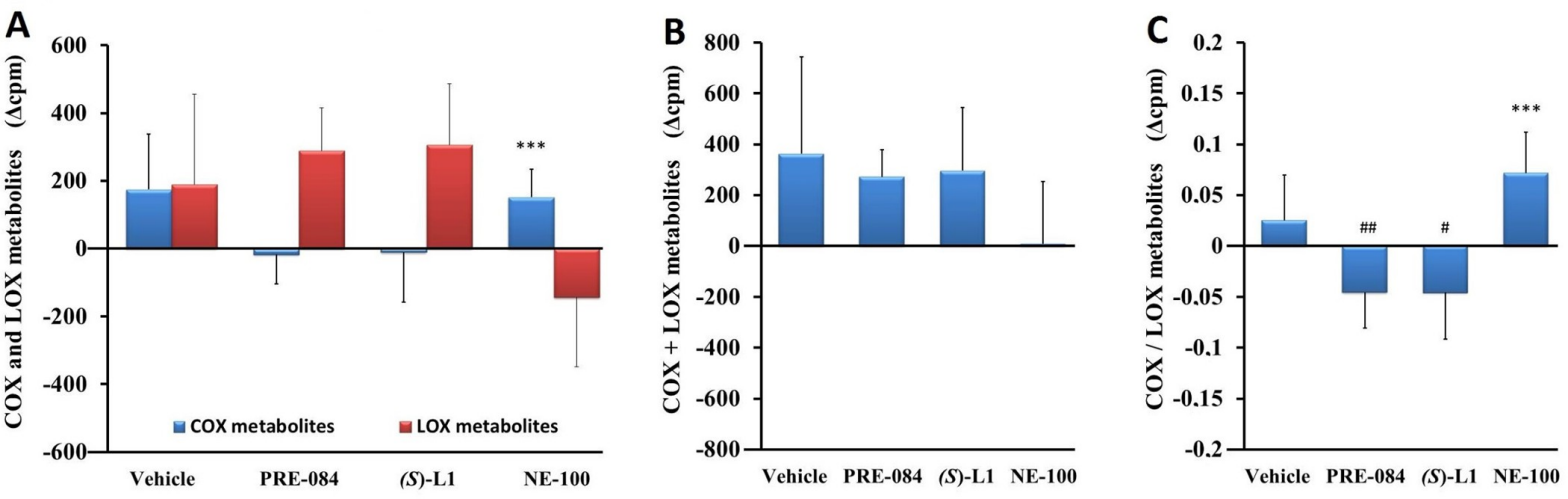
Arachidonic acid metabolism in rat platelets. COX and LOX AA metabolites (A), total quantity of AA metabolites (COX+LOX) (B) and the ratio of COX to LOX AA metabolites (COX/LOX) (C) in the platelets from the diabetic vehicle, PRE-084, *(S)-*L1 or NE-100-treated rats are shown as compared to the vehicle-treated, healthy animals. The zero line in the diagram shows the mean for the isotope activity of the vehicle-treated, healthy rat platelets, while the columns in the figure represent the delta mean±SD value of the diabetic treatment group; n = 9 samples per group; Welch’s ANOVA, Dunnett’s T3 test, ***p<0.01 compared to the vehicle-treated, healthy rats group; ^#^p<0.05, ^##^p<0.03 compared to the vehicle-treated, diabetic rat group; cpm: count per minute; COX: total quantity of cyclooxygenase metabolites synthesized from AA in platelets; LOX: total quantity of lipoxygenase metabolites synthesized from AA in platelets.

*Platelet eicosanoid synthesis from radioactive AA substrate*. In the platelets, one month after the development of STZ-induced diabetes, neither the total quantity of COX metabolites (the sum of 6-keto prostaglandin (PG) F_1α_ (6-k-PGF_1α_), which is a stable metabolite of prostacyclin; PGF_2α_; PGE_2_; PGD_2_; thromboxane B_2_ (TxB_2_), which is a stable metabolite of TxA_2_ and 12-L-hydroxy-5,8,10-heptadecatrienoic acid), nor the total quantity of eicosanoids formed by the LOX pathway was altered (Fig 3A) compared to healthy rats.

In the diabetic rat platelets, the total quantity of the COX metabolites was significantly eleveted by NE-100 compared to the vehicle-treated, healthy rat platelets. The total quantity of the AA metabolites (COX+LOX) in the diabetic platelets was not significantly modified by the S1R ligands compared to the vehicle-treated, healthy and diabetic samples (Fig 3B**)**. Although there was no detectable change in platelet COX/LOX ratio one month after the STZ-induced development of diabetes, treatment with PRE-084 or *(S)*-L1 significantly reduced it compared to the vehicle-treated, diabetic samples. Meanwhile, NE-100 increased the COX/LOX ratio of the diabetic platelets as compared to the vehicle-treated, healthy rat platelets (Fig 3C).

**Fig 4:**
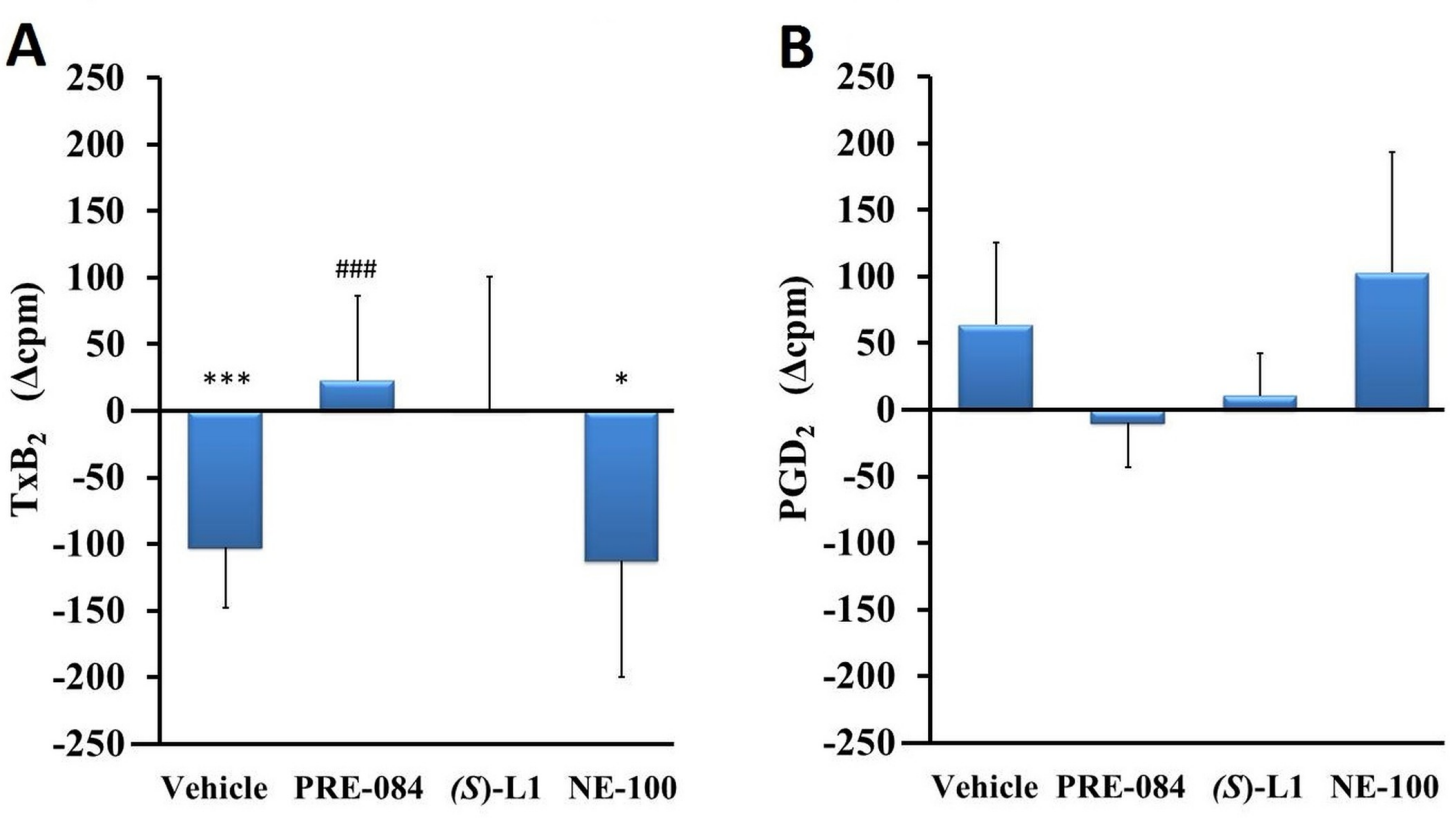
The effects of S1R ligands on the different COX metabolites in rat platelets. TxB_2_ (A) and PGD_2_ (B) COX metabolites in the platelets from diabetic vehicle, PRE-084, *(S)-*L1 or NE-100-treated rats are shown as compared to the vehicle-treated, healthy animals. The zero line in the diagram shows the mean for the isotope activity of the vehicle-treated, healthy rat platelets, while the columns in the figure represent the delta mean±SD values for the diabetic treatment groups; n = 9 samples per group; Welch’s ANOVA, Dunnett’s T3 test, *p<0.05, ***p<0.01 compared to the vehicle-treated, healthy rat group; ^###^p<0.01 compared to the vehicle-treated, diabetic rat group; cpm: count per minute; TxB_2_: thromboxane B_2_; PGD_2_: prostaglandin D_2_.

In the platelets from the vehicle-treated, diabetic rats, the synthesis of TxB_2_ was significantly decreased (Fig 4A), while the production of PGD_2_ (Fig 4B), vasodilator and platelet anti-aggregator product was not significantly changed compared to the healthy animals (Fig 4).

PRE-084 elevated the synthesis of TxB_2_ in diabetic platelets compared to the vehicle treated, diabetic rat platelets, while NE-100 reduced it compared to the vehicle-treated, healthy rat platelets (Fig 4A).

**Fig 5:**
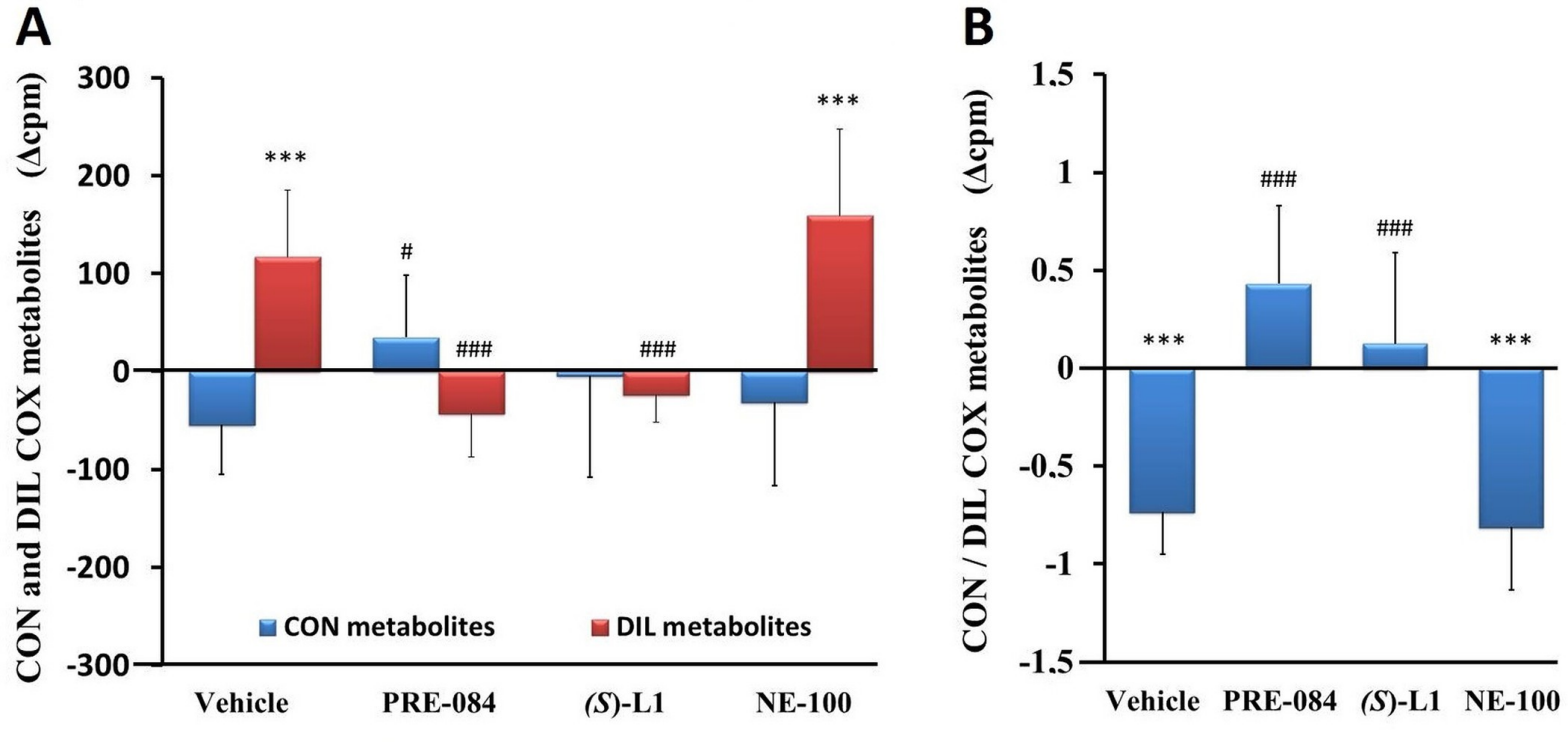
Vasoconstrictor, platelet aggregator (CON) and vasodilator, platelet anti-aggregator (DIL) COX metabolites of rat platelets. CON COX and DIL COX AA metabolites (A) and the ratio of CON and DIL COX metabolites (CON/DIL) (B) in the platelets from the diabetic vehicle, PRE-084, *(S)-*L1, or NE-100-treated rats are shown as compared to the vehicle-treated, healthy animals. The zero line in the diagram shows the mean for the isotope activity of the vehicle-treated, healthy rat platelets, while the columns in the figure represent the delta mean±SD values for the diabetic treatment groups; n = 9 samples per group; Welch’s ANOVA, Dunnett’s T3 test, ***p<0.01 compared to the vehicle-treated, healthy rat group; ^#^p<0.05, ^###^p<0.01 compared to the vehicle-treated, diabetic rat group; cpm: count per minute; CON: the sum of PGF_2α_ and TxB_2_; DIL: the sum of PGE_2_ and PGD_2_.

In the vehicle-treated, diabetic rat platelets, the synthesis of the vasoconstrictor and platelet aggregator COX metabolites (CON: the sum of PGF_2α_ and TxB_2_) was not changed significantly, while the quantity of vasodilator and anti-aggregator COX products (DIL: sum of PGE_2_ and PGD_2_) was increased (Fig 5A) compared to the healthy rat platelets.

The quantity of DIL COX metabolites in the diabetic platelets treated with NE-100 was significantly higher (Fig 5A) and consequently the ratio of CON to DIL COX metabolites was lower than in the healthy platelets from the vehicle-treated rats (Fig 5B). The quantity of the CON COX metabolites was raised in the diabetic platelets by the ligand PRE-084 as compared to the vehicle-treated, diabetic platelets. The synthesis of the DIL COX metabolites was reduced not only by PRE-084, but also by *(S)*-L1 compared to the vehicle-treated, diabetic group (Fig 5A). The CON/DIL ratio was significantly lower in the vehicle-treated, diabetic rat platelets than in the vehicle-treated, healthy ones (Fig 5B). In the platelets in the diabetic rats, an increase in CON/DIL ratio was observed in both the PRE-084 and *(S)*-L1-treated groups compared to the vehicle-treated, diabetic rat platelets (Fig 5B)

**Fig 6:**
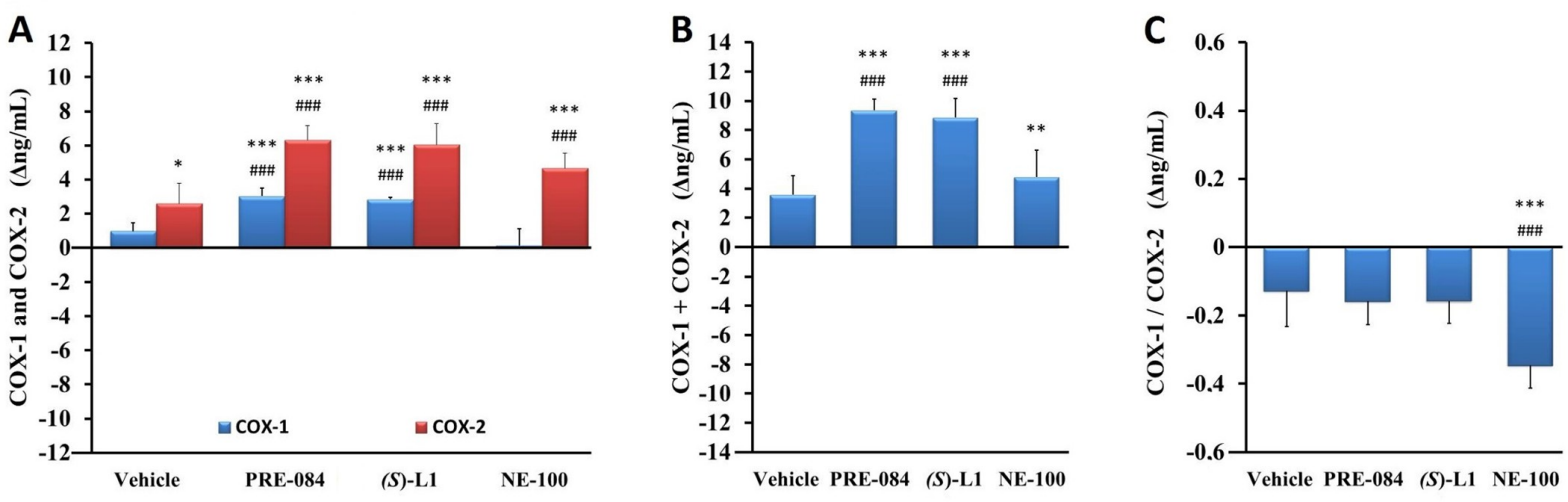
Effect of S1R ligands on the quantity of COX-1 and COX-2 enzymes in rat platelets as determined by ELISA. Level of COX-1 and COX-2 enzymes (A), the total quantity of COX enzymes (COX-1+COX-2) (B) and the ratio of COX-1 to COX-2 (COX-1/COX-2) (C) in the platelets from the diabetic vehicle, PRE-084, *(S)-*L1 or NE-100-treated rats are shown as compared to the vehicle-treated, healthy animals. The zero line in the diagram shows the mean for the concentration of COX enzymes of the vehicle-treated, healthy rat platelets, while the columns in the figure represent the delta mean±SD values for the diabetic treatment groups; n = 9 samples per group; Welch’s ANOVA, Dunnett’s T3 test, **p<0.02, ***p<0.01 compared to the vehicle-treated, healthy rat group; ^###^p<0.01 compared to the vehicle-treated, diabetic rat group; COX-1: Type 1, constitutive cyclooxygenase; COX-2: Type 2, inducible cyclooxygenase.

*COX enzyme levels in rat platelets as determined by ELISA*. The concentration of constitutive COX-1 in the vehicle-treated, diabetic rat platelets was not different, whereas the concentration of inducible COX-2 was significantly higher than in the vehicle-treated, healthy platelets (Fig 6A).

In the diabetic rat platelets, the concentration of COX-1 was increased by the ligands PRE-084 and *(S)*-L1, while the COX-2 level was raised by all three S1R ligands as compared to both the vehicle-treated, healthy and diabetic rats (Fig 6A). The total quantity of COX enzymes (COX-1+COX-2), was also elevated by all three S1R ligands, but in the case of the NE-100 ligand this was due to rise in COX-2 levels (Fig 6B). This is supported by a decrease in the COX-1/COX-2 ratio of the rat platelets treated with the NE-100 ligand compared to the vehicle-treated, healthy and diabetic samples (Fig 6C).

#### Effects of S1R ligands on the *ex vivo* eicosanoid synthesis of the diabetic rat abdominal aorta

**Fig 7:**
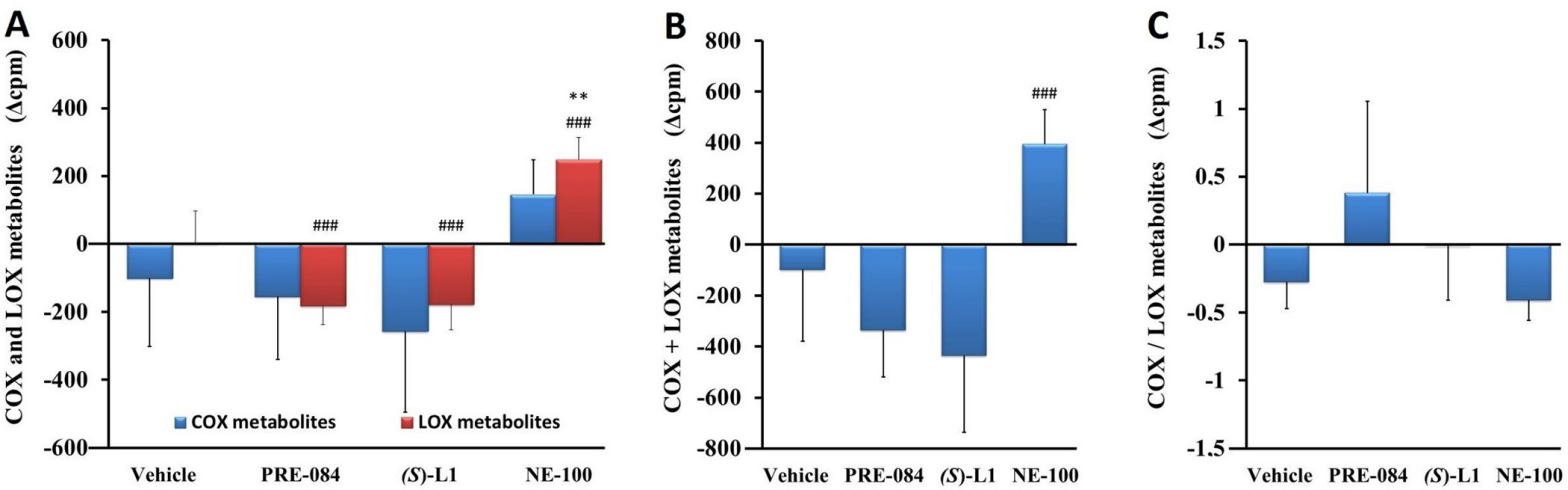
Arachidonic acid metabolism in rat abdominal aorta. COX and LOX AA metabolites (A), total quantity of AA metabolites (COX+LOX) (B), and the ratio of COX to LOX AA metabolites (COX/LOX) (C) synthesized in the aorta of the diabetic vehicle, PRE-084, *(S)*-L1 or NE-100-treated rats are shown as compared to the vehicle-treated, healthy animals. The zero line in the diagram shows the mean for the isotope activity of the vehicle-treated, healthy rat aorta, while the columns in the figure represent the delta mean±SD value of the diabetic treatment groups; n = 9 samples per group; Welch’s ANOVA, Dunnett’s T3 test, **p<0.03 compared to the vehicle-treated, healthy rat group; ^###^p<0.01 compared to the vehicle-treated, diabetic rat group; cpm: count per minute; COX: total quantity of cyclooxygenase metabolites synthesized from AA in abdominal aorta; LOX: total quantity of lipoxygenase metabolites synthesized from AA in abdominal aorta.

*Aortic eicosanoid synthesis from radioactive AA substrate*. The total quantity of metabolites synthesized from AA by COX or LOX enzymes in the aorta samples of the vehicle-treated, diabetic animals did not differ significantly from that of the vehicle-treated, healthy rats (Fig 7A).

The abdominal aortic AA metabolism (COX+LOX metabolites) (Fig 7B) and COX/LOX product ratio of the vehicle-treated, diabetic rat (Fig 7C) did not change compared to the vehicle-treated, healthy animal. The synthesis of LOX metabolites was significantly increased in the NE-100-treated, diabetic rat abdominal aorta compared to the vehicle-treated, healthy or diabetic one. PRE-084 and *(S)*-L1 administration reduced the production of LOX metabolites, without modifying the COX pathway in the diabetic aorta compared to the vehicle-treated, diabetic one (Fig 7A). The quantity of AA metabolites (COX+LOX) in the diabetic rat abdominal aorta was raised in the NE-100-treated group compared to the vehicle-treated, diabetic animals (Fig 7B). The COX/LOX metabolite ratio in the diabetic rat aorta was not modified by any of the S1R ligands compared to the vehicle-treated, healthy or diabetic rats (Fig 7C).

**Fig 8:**
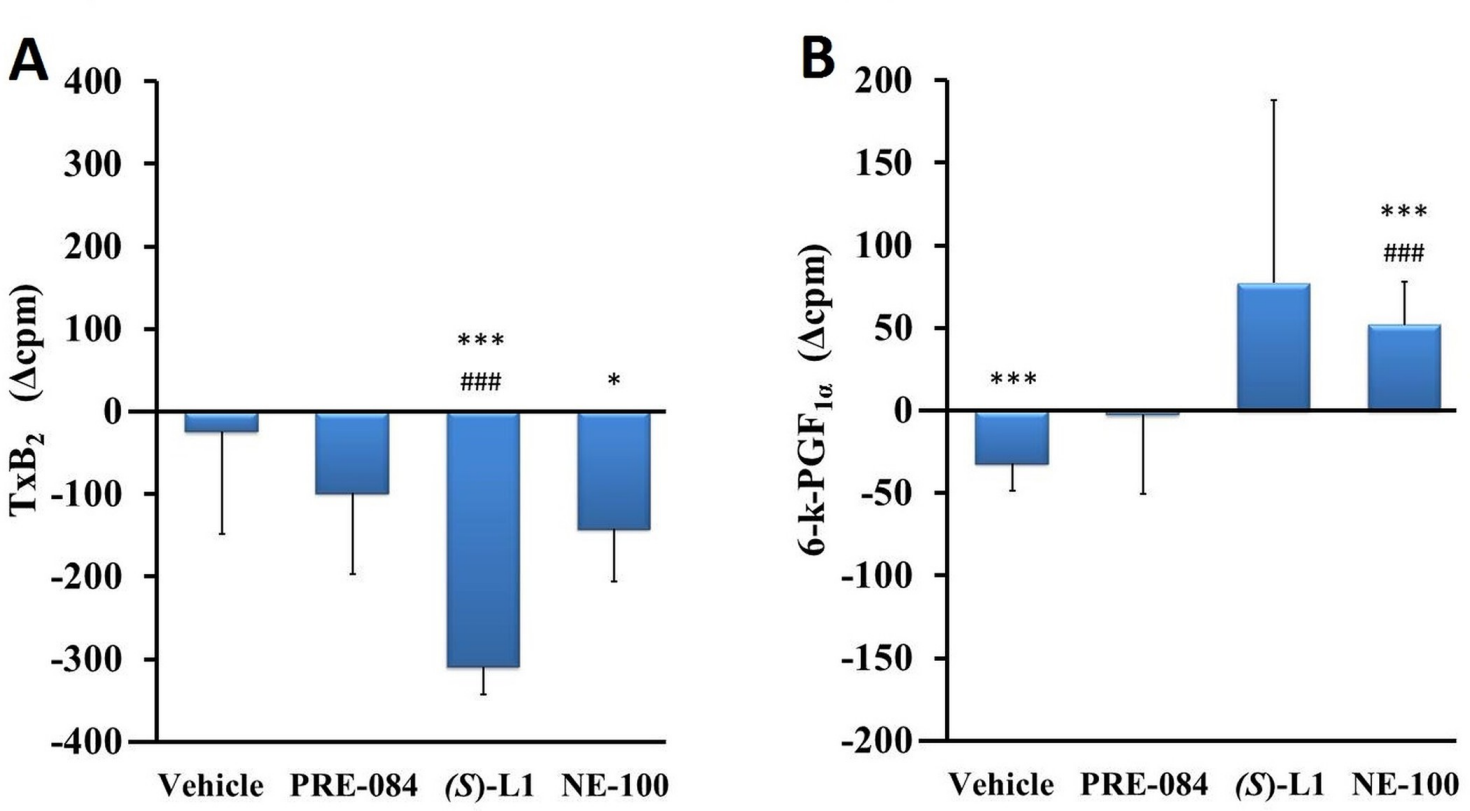
The effects of S1R ligands on the different COX metabolites in the rat aorta. TxB_2_ (A) and 6-k-PGF_1α_ (B) COX metabolites synthesized in the aorta of diabetic vehicle, PRE-084, *(S)*-L1 or NE-100-treated rats are shown as compared to the vehicle-treated, healthy animals. The zero line in the diagram shows the mean for the isotope activity of the vehicle-treated, healthy, rat aorta, while the columns in the figure represent the delta mean±SD values for the diabetic treatment groups; n = 9 samples per group; Welch’s ANOVA, Dunnett’s T3 test, *p<0.05, ***p<0.01 compared to the vehicle-treated, healthy rat group; ^###^p<0.01 compared to the vehicle-treated, diabetic rat-group; cpm: count per minute; TxB_2_: thromboxane B_2_; 6-k-PGF_1α_: 6-keto prostaglandin PGF_1α_.

The synthesis of the vasoconstrictor TxB_2_ was not changed (Fig 8A), while the production of 6-k-PGF_1α_, the main vasodilator and platelet anti-aggregator product of the aorta, was decreased significantly in the vehicle-treated, diabetic rat aorta (Fig 8B) compared to the healthy animals.

*(S)*-L1 induced the reduction of TxB_2_ synthesis in the diabetic aorta compared to the vehicle-treated, healthy and diabetic ones (Fig 8A). The NE-100 ligand had opposite effects on these eicosanoids in the diabetic rat abdominal aorta: it reduced the production of TxB_2_ compared to the vehicle-treated, healthy rats (Fig 8A) and increased the synthesis of 6-k-PGF_1α_ compared to the vehicle-treated, healthy and diabetic rats (Fig 8B).

**Fig 9:**
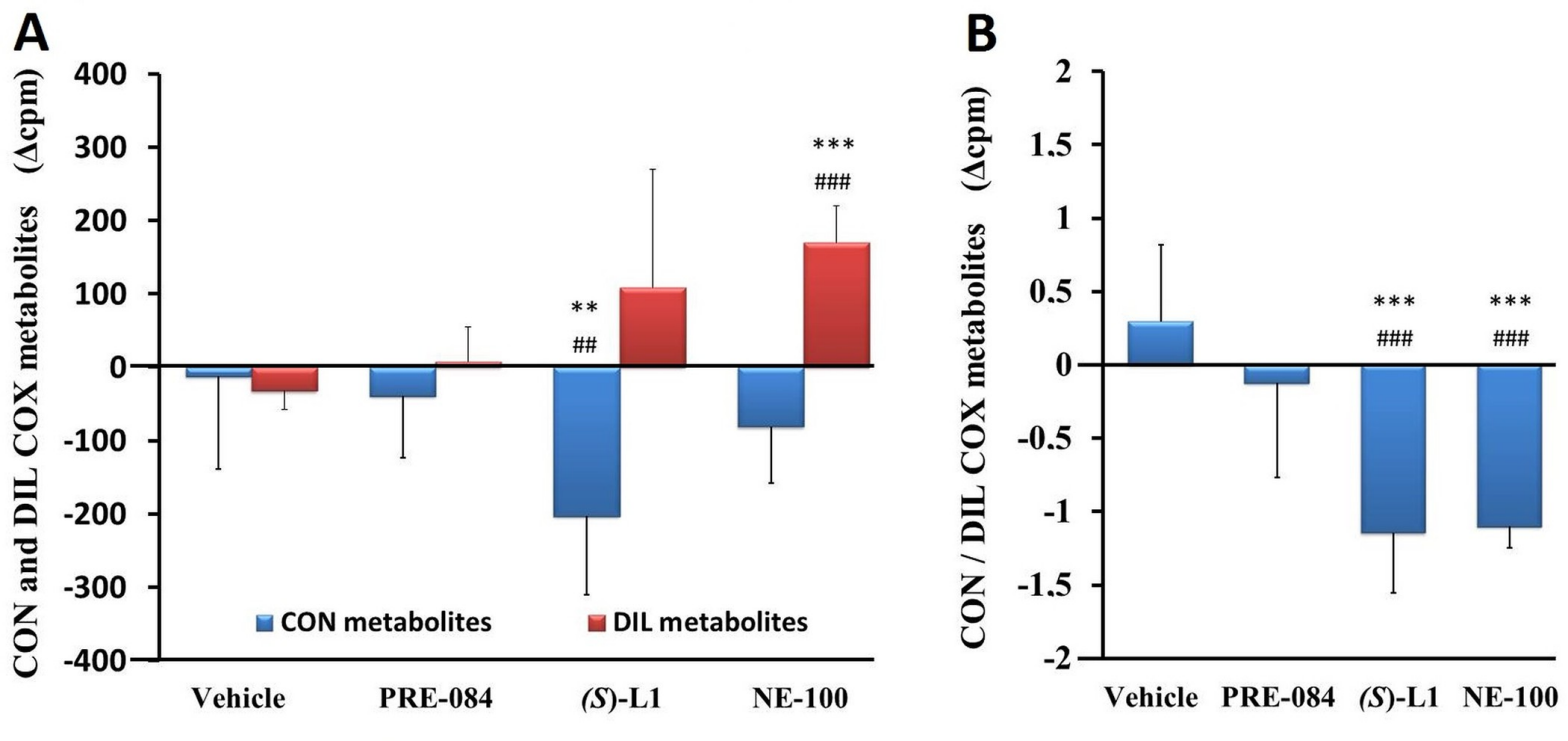
Vasoconstrictor, platelet aggregator (CON) and vasodilator, platelet anti-aggregator (DIL) COX metabolites of rat abdominal aorta. CON COX and DIL COX metabolites (A) and the ratio of CON to DIL COX metabolites (CON/DIL) (B) synthesized in the aorta of the diabetic vehicle, PRE-084, *(S)*-L1 or NE-100-treated rats are shown as compared to the vehicle-treated healthy animals. The zero line in the diagram shows the mean for the isotope activity of the vehicle-treated, healthy rat aorta, while the columns in the figure represent the delta mean±SD values for the diabetic treatment groups; n = 9 samples per group; Welch’s ANOVA, Dunnett’s T3 test, **p<0.03, ***p<0.01 compared to the vehicle-treated, healthy rat group; ^##^p<0.03, ^###^p<0.01 compared to the vehicle-treated, diabetic rat group; cpm: count per minute; CON: the sum of PGF_2α_ and TxB_2_; DIL: the sum of 6-k-PGF_1α_ (that is a stable metabolite of prostacyclin), PGE_2_ and PGD_2_.

No alterations in CON COX and DIL COX metabolite were detected in the abdominal aorta of the vehicle-treated, diabetic rats (Fig 9A) compared to the aorta of the vehicle-treated, healthy animals.

The production of CON COX metabolites of the diabetic rat aorta was significantly reduced by *(S)*-L1 (Fig 9A) compared to the vehicle-treated, healthy and diabetic groups. The synthesis of DIL COX metabolites in the diabetic rat abdominal aorta was stimulated by NE-100 compared to the vehicle-treated, healthy and diabetic samples (Fig 9A). Both the *(S)*-L1 and the NE-100 ligands significantly decreased the CON/DIL ratio in the diabetic rat abdominal aorta ring compared to the vehicle-treated, healthy and diabetic samples (Fig 9B).

**Fig 10:**
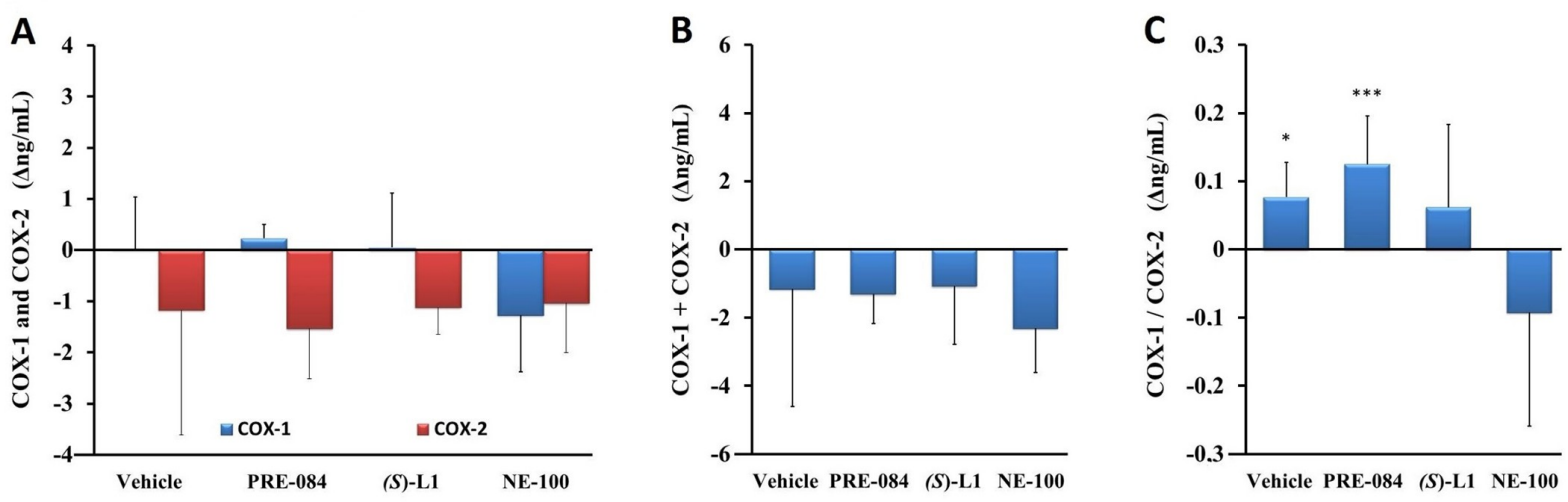
Effect of S1R ligands on levels of COX-1 and COX-2 enzymes determined by ELISA in rat abdominal aorta. Enzyme concentrations of COX-1 and COX-2 (A), the total quantity of COX enzymes (COX-1+COX-2) (B) and the ratio of COX-1 to COX-2 (COX-1/COX-2) (C) in the aorta of the diabetic vehicle, PRE-084, *(S)*-L1 or NE-100-treated rats are shown as compared to the vehicle-treated, healthy animals. The zero line in the diagram shows the mean for the enzyme concentration of the vehicle-treated, healthy rat aorta, while the columns in the figure represent the delta mean±SD values for the diabetic treatment groups; n = 9 samples per group; Welch’s ANOVA, Dunnett’s T3 test, *p<0.05, ***p<0.01 compared to the vehicle-treated healthy rat group; ^#^p<0.05 compared to the vehicle-treated diabetic rat group; COX-1: Type 1 cyclooxygenase; COX-2: Type 2 cyclooxygenase.

*COX enzyme levels in the abdominal rat aorta measured by ELISA*. The concentration of COX-2 in the aortas of both healthy and diabetic vehicle-treated rats was higher than that of COX-1. Neither the concentration of COX-1 or COX-2 (Fig 10A) nor their sum (COX-1+COX-2) (Fig 10B) in the aorta of the vehicle-treated, diabetic rats showed significant differences compared to vehicle-treated, healthy animals.

In the aorta of diabetic rats, the concentrations of these parameters were not altered by any of the S1R ligands we tested (Fig 10A, 10B). However, the COX-1/COX-2 ratio in the aorta of diabetic rats treated with vehicle or PRE-084 was significantly higher than in healthy rats treated with vehicle (Fig 10C).

## Discussion

In the present study, we investigated the effects of sub-chronic, *in vivo* administered S1R ligands on *ex vivo/in vitro* eicosanoid synthesis of STZ-induced diabetic rat platelets and abdominal aorta. We selected STZ-induced diabetes mellitus as a reliable, reproducible model with chronic inflammation and oxidative stress, in which the development and time course of complications can be easily monitored [35]. STZ contains glucose and N-methyl-N-nitrosocarbamide groups [36]. Its glucose component binding to glucose transporter-2 (GLUT-2) promotes the entry of STZ into pancreatic, liver and renal tubule epithelial cells with GLUT-2 [37, 38]. The N-methyl-N-nitrosourea constituent of STZ thus transported into cells induces DNA methylation [39], alkylation [40] and oxidation [41]. These processes induce cell apoptosis, leading to the development of diabetes mellitus and liver and kidney damage.

In the present study, the development of STZ-induced diabetes mellitus was supported by elevated serum glucose level (hyperglycaemia) and a reduced rate of body weight gain in the rats compared to the physiological parameters. None of the ligands significantly altered the STZ-induced increase in serum glucose concentration and decreased body weight gain (Table 1), indicating they were unable to restore the STZ-induced metabolic changes. Consistent with our previous study [42], we found elevated serum cholesterol, ALT and urea levels in the STZ-induced diabetic animals, which may be explained by the hepatic and renal toxic effects of STZ [37, 38]. The STZ-induced rise in serum ALT concentration was further boosted by the S1R ligands, although the growth was not significant for PRE-084 (Fig 1). These results suggest the metabolism and/or elimination of ligands in the liver or kidney [43]. The platelet count below the reference value [44], already observed in our previous study, can be explained by reduced thrombopoietin production due to the liver and kidney damage associated with STZ-induced diabetes [42]. However, in the present study, we only observed a trend towards a decrease in platelet counts in the STZ-treated animals compared to the vehicle-treated, healthy rat group, which was normalized by PRE-084 and NE-100. The slightly reduced platelet number in diabetic rats does not influence the results of the present study, as eicosanoid synthesis was monitored at a standard platelet count (Table 1).

Ha et al. (2012) reported a protective role of S1R against pathological changes in STZ-induced diabetes mellitus [24]. Since both platelets and endothelial cells are involved in the development of diabetic complications [45], we hypothesized that S1R ligands could modulate the function of both cell types. We selected PRE-084, NE-100 and *(S)*-L1 from among the S1R ligands based on their binding strength to the receptor and their binding site in the S1R binding pocket as determined in our previous experiments [19]. Based on platelet lifespan, the duration of *in vivo* treatment was one week [46, 47]. Preliminary time-dependent studies of the serum levels of i.p. administered S1R ligands demonstrated that all ligands entered the circulation. However, their serum concentrations were only at the limit of detection 20 h after the last treatment; that is, it was possible to rule out the direct acute effect of the ligands during the *ex vivo* experiment. Thus, the *ex vivo* changes in platelet and abdominal aortic AA metabolism were attributed to the *in vivo* effects of S1R ligands on platelet and aortic function [19].

Sub-chronic, *in vivo* treatment with S1R ligands was also able to alter the AA metabolism of the platelets and aorta in the STZ-induced diabetic rats despite the absence of the ligands in the blood at the time of the *ex vivo/in vitro* study (Figs 3 and 7). S1R ligands can modulate the PL content of the cell membrane, the re- or de-acylation of PLs, and the amount of free AA released from PLs by phospholipases [4, 48, 49]. They may also affect the expression and/or activity of COX and LOX enzymes involved in AA metabolism. From the two COX isoforms, COX-1 is constitutively expressed in various tissues, but the expression of COX-2 is mostly inducible [50]. Although the platelets are anucleated cells, they can *de novo* synthesize COX-1 from the cytoplasmic mRNA that originates from the megakaryocyte [51]. In human platelets, Hu and co-workers [52] also detected COX-2 mRNA and protein, although at significantly lower levels than COX-1, consistent with our data. However, under the current experimental conditions, we did not detect *Ptgs2* mRNA in the platelets from the healthy control [19] or diabetic rats with RT-qPCR. This may be explained by the fact that only limited amounts of megakaryocyte mRNA transcripts are available for protein synthesis (e.g. COX-2), and therefore, *in vivo* AA metabolism in platelets may lead to depletion of intracellular reserves (mRNA, enzyme pool and ☯Ca^2+^]_i_). Unchanged *Sigmar1* and increased *Ptgs1* transcript levels were detected in the platelets of the vehicle-treated, diabetic rats compared to the healthy ones. Based on these results, our hypothesis that platelets from the vehicle-treated, diabetic rats raised *Sigmar1* transcript expression was not confirmed. However, under pathological conditions, S1R agonist PRE-084 with antioxidant properties [53] as well as the novel SR-1 ligand *(S)*-L1 were able to enhance S1R transcript level (Fig 2), which may have a protective role against oxidative stress in diabetes. NE-100 decreased *Ptgs1* compared to the vehicle-treated, diabetic group. These results support our hypothesis that PRE-084 S1R agonist and NE-100 antagonist have opposite effects on platelet *Sigmar1* and *Ptgs1* transcript levels. The structural similarity between *(S)*-L1 and NE-100 is only reflected in the similar effect on platelet *Ptgs1* levels, suggesting that the modulatory effect of S1R ligands is enzyme-, cell- and tissue-specific.

A rise in DIL COX products and a drop in the CON/DIL ratio were observed in the diabetic rat platelets (Fig 5), compared to the healthy animals, while a drop in 6-k-PGF_1α_ synthesis was detected in aorta (Fig 8). These results support our hypothesis that platelet and endothelial cell activation *in vivo* can be detected *ex vivo*. In the platelets we observed a change opposite to that expected, which could be the result of a smaller storage pool of platelets (e.g. intracellular calcium ion) during *in vivo* platelet activation or a consequence of a compensatory mechanism that develops early in diabetes. In our previous *ex vivo* platelet aggregation study in STZ-induced diabetic rats, no increased platelet aggregation capacity was measured in diabetic rats either [42]. Consistent with the present data, decreased *in vitro* ADP- and AA-induced platelet aggregation was found in the STZ-induced diabetic rats compared to the healthy animals [54]. Paradoxically, these effects paralleled the high intrinsic hyperactivity of the platelets and might be explained by the time-dependent production of platelet aggregation-inhibiting factors.

Treatment with the ligands PRE-084 and *(S)*-L1 did not affect the total quantity of the radioactive eicosanoids (COX+LOX) synthesized by the platelets from the diabetic rats, but it lowered the COX/LOX ratio. These results suggest that PRE-084 and *(S)*-L1 induce a shift in platelet AA metabolism towards the LOX pathway without altering the amount of AA substrate released from PLs (Fig 3).

Although PRE-084 and *(S)*-L1 did not elevate COX-1 mRNA (Fig 2) in the diabetic platelets compared to the vehicle-treated diabetic platelets, they raised COX enzyme concentrations as determined with ELISA (Fig 6). While this did not induce a rise in the total quantity of COX pathway products (Fig 3), i.e. an increase in COX enzyme activity, it modulated the function of the enzymes involved in the synthesis of individual COX products to normalize the changes measured in diabetic platelets (Figs 4, 5). For example, in diabetic rats, a higher CON/DIL ratio was observed in the platelets in the PRE-084 and *(S)*-L1 treatment groups, indicating a predominance of CON COX metabolites (Fig 3) compared to platelets from the vehicle-treated, diabetic rats, which, in contrast, showed a reduced CON/DIL ratio compared to the vehicle-treated, healthy group. NE-100 did not affect the AA metabolism of platelets from diabetic animals compared to the vehicle-treated, diabetic group (Figs 3–6).

Although several safety procedures have been taken to prevent spontaneous aggregation of platelets during platelet isolation and in vitro studies, and no direct platelet aggregation was induced in ex vivo/in vitro studies, the possibility of activation of platelet intracellular signalling pathways cannot be completely excluded. EDTA used during platelet separation reduced the amount of extracellular calcium ion available to platelets, the calcium ion level was normalized by the tissue culture medium used in the ex vivo/in vitro study. In our ex vivo/in vitro study, as the tissue culture medium did not contain fibrinogen, platelet aggregation could not occur. We consider a limitation of our study that we have not studied a population of platelets at rest, but a population of platelets adapted to changes in the culture medium environment with physiological parameters. However, this does not make it impossible to compare the effects of S1R ligands ex vivo, as the ligand-treated and non-ligand-treated samples were tested in parallel, from the same platelet population.

The effects of S1R ligands on eicosanoid synthesis in abdominal aorta were very different from those observed in the platelets. Reduced synthesis of 6-k-PGF_1α_ was detected in the aorta of diabetic rats (Fig 8). In the aorta, NE-100 was found to be the most potent S1R ligand in promoting the recovery of physiological status by enhancing 6-keto-PGF_1α_ synthesis. The increase in the total quantity of AA metabolites (COX+LOX) in the NE-100 group (Fig 7) suggests that this is likely to be due not only to specific prostacyclin synthase, but also to the effect of higher AA substrate due to phospholipase activation. (S)-L1, in turn, increases the rate of vasodilator COX products (Fig 9) by decreasing the synthesis of vasoconstrictor, platelet aggregator eicosanoids compared to both vehicle-treated, diabetic and vehicle-treated, healthy rat aorta. In the aortic AA metabolism study, PRE-084 was the most ineffective ligand, not affecting the synthesis of COX pathway products compared to either vehicle-treated, healthy or vehicle-treated, diabetic rats. Based on these results, our hypothesis that S1R ligands applied sub-chronically *in vivo* can modulate both platelet and aortic AA metabolism *ex vivo* was confirmed. The AA metabolism of platelets and endothelial cells are known to differ significantly under healthy conditions. Platelets primarily synthesize the vasoconstrictor thromboxane, whereas endothelial cells primarily synthesize the vasodilator prostacyclin. The balance between these arachidonic acid metabolites plays a crucial role in maintaining normal local circulation [31, 39, 42]. Overall, S1R ligands have opposite effects on platelet and aortic AA metabolism in diabetic rats. Our hypothesis that S1R ligands modulate the pathological AA metabolism in diabetic rat platelets and aortic rings in such a way that the physiological balance between them is restored was confirmed.

### Conclusion

In STZ-induced diabetic rats platelet TxB_2_ and aortic 6-k-PGF_1α_ production dropped. Sub-chronic *in vivo* treatment of diabetic animals with the PRE-084 ligand enhanced CON COX and reduced DIL COX product formation, while *(S)*-L1 lowered the synthesis of DIL COX metabolites and promoted the recovery of physiological platelet function in diabetic rats. The S1R antagonist NE-100, on the other hand, produced no significant changes in the diabetic rat platelet AA metabolism. In contrast, *(S)*-L1 decreased the synthesis of CON COX metabolites, whereas NE-100 increased the quantity of aortic DIL COX products and promoted the recovery of diabetic endothelial dysfunction in the aorta. S1R agonist PRE-084 did not change aortic AA metabolism. The novel S1R ligand *(S)*-L1 had effects on eicosanoid synthesis in platelets similar to those of the agonist PRE-084 and on eicosanoid synthesis in aorta similar to those of the antagonist NE-100. Our results suggest that S1R ligands may play a role in the regulation of cellular functions and local circulation by affecting AA metabolism at transcription, translation and enzyme induction levels. In diabetes mellitus, the cell-specific effects of S1R ligands have a compensatory role and aid in restoring physiological balance between the platelet and vessel.

## Supporting information

S1 Appendix Experimental animal groups. (PDF)

S2 Appendix Timeline of experiments. (PDF)

S3 Appendix Sampling protocol. (PDF)

S1 Graphical abstract (TIF)
