# Supplementary material for: Effects of sub-chronic, in vivo administration of sigma-1 receptor ligands on platelet and aortic arachidonate cascade in streptozotocin-induced diabetic rats
Source: PLoS One. 2022 Nov 17;17(11):e0265854. doi: 10.1371/journal.pone.0265854 (PMC9671357; doi:10.1371/journal.pone.0265854)
Supplement: S1 Appendix — (PDF) [file pone.0265854.s001.pdf]

Supplementary information on the experimental animal groups

The sub-chronic, *in vivo* effects of S1R ligands involved a total of 81 *male Wistar* rats *(Rattus norvegicus)*. Inclusion in the experiments was based on good health, normal serum glucose level, age-appropriate food and fluid intake, and body weight. All 81 animals fulfilled these criteria, so exclusion from the study was not necessary (Schulz et al., 2010).

After weaning, three rats were placed in a transparent-walled cage. These housing conditions were used to reduce the isolation and environmental discomfort caused by diabetes symptoms in rats. As required by guidelines and the ethics permit, we placed enrichment devices (e.g. cylinders and cubes) in the rat cages. After a week of adaptation to the environment and handling, they were divided into two groups.

In our preliminary experiment, we used nine animals (three animals/group) selected from this animal population by simple randomization (Urbaniak and Plous, 2013) to investigate blood levels of S1R ligands (PRE-084, *(S)*-L1, NE-100) in a time-dependent manner following the administration of a single intraperitoneal injection of 3 mg/bw kg (see Fig S1).

The remaining 72 rats were used to study the *in vivo* effects of S1R ligands on platelet and aortic arachidonic acid metabolism. The animals were divided into two groups by simple randomization. One of the resulting groups of 36 rats was the non-diabetic or healthy population while the other group consisted of animals who became diabetic after treatment with streptozotocin (See the "Diabetes animal model" section of the manuscript). Healthy and diabetic animal populations of 36 rats each (15 weeks old) were allocated into subgroups of 9 rats each by simple randomization for treatment with vehicle or one of the S1R ligands (PRE-084 or *(S)*-L1 or NE-100, 3 mg/bw kg, i.p.) as shown in Fig S1. Criteria for inclusion in the subpopulation were physiologically adequate daily food and fluid intake and maintenance of initial body weight.

81 male Wistar *(Rattus norvegicus)* rats
Total number of rats eligible for inclusion in the present study

↓ ↓

3 x 3 = 9 rats

for ***preliminary experiment***
for determination of time-dependent
plasma levels of i.p. injected S1R ligands
(Váczi et al. 2022)

72 rats

for ***examination of in vivo effect***
of S1R ligands on arachidonic
acid metabolism

↓

in parallel with each other

↓ ↓

Non-diabetic, healthy rats
Total number of treated animals is 36

vehicle	PRE-084	*(S)*-L1	NE-100
**9 rats**	9 rats	9 rats	9 rats

(Váczi et al. 2022)

Diabetic rats
Total number of treated animals is 36

**vehicle**	**PRE-084**	***(S)*-L1**	**NE-100**
**9 rats**	**9 rats**	**9 rats**	**9 rats**

(Data presented in this manuscript)

Fig S1 Schematic drawing of the experimental setup, treatment groups and number of animals in each group

References

Urbaniak, G. C., Plous S. Research Randomizer (Version 4.0) Computer software [Internet]. 2013 [cited 2022 Jul 28]. Available from: https://www.randomizer.org/

Schulz KF, Altman DG, Moher D. CONSORT 2010 statement: Updated guidelines for reporting parallel group randomised trials. J Pharmacol Pharmacother. 2010 Jul;1(2):100-7.

Váczi S, Barna L, Laczi K, Tömösi F, Rákhely G, Penke B, Fülöp L, Bogár F, Janáky T, Deli MA, Mezei Z. Effects of sub-chronic, in vivo administration of sigma non-opioid intracellular receptor 1 ligands on platelet and aortic arachidonate cascade in rats. Eur J Pharmacol. 2022 Jun 15;925:174983.
