# Supplementary material for: Effects of sub-chronic, in vivo administration of sigma-1 receptor ligands on platelet and aortic arachidonate cascade in streptozotocin-induced diabetic rats
Source: PLoS One. 2022 Nov 17;17(11):e0265854. doi: 10.1371/journal.pone.0265854 (PMC9671357; doi:10.1371/journal.pone.0265854)
Supplement: S2 Appendix — (PDF) [file pone.0265854.s002.pdf]

ADDITIONAL INFORMATION ON THE TIMELINE FOR THE EXPERIMENTS ON THE DIABETIC RATS

1. Selection of male Wistar (*Rattus norvegicus*) rats according to the criteria for inclusion in the experiment, after weaning

2. Simple randomization of rats

3. Three rats from the same group were placed in a transparent-walled cage

4. One week of adaptation to the environment and handling

5. Basal body weight and blood sugar determination

6. Induction of diabetes mellitus by a single i.p. injection of STZ (65 mg/ kg body weight)

7. Replacement of animals' drinking water with a 10% (w/v) sucrose solution for 24 hours

8. Regular, twice daily health check on the condition of the animals

9. Initial body weight and blood sugar determination 72 hours after the STZ injection

10. Simple randomization of rats to create groups of animals for treatment

11. Start of treatment (once a day for a week) with S1R ligands PRE-084 or (S)-L1 or NE-100 (3 mg/ kg body weight i.p.) or vehicle (0.9% sodium chloride)

12. Twenty hours after the last treatment:
    - Determination of terminal body weight
    - Blood sampling under anaesthesia

13. After sampling, rats were euthanized with pentobarbital (100 mg/ kg body weight i.p.)

14. Collection of abdominal aorta samples

15. Determination of terminal parameters: serum ligand levels, laboratory data

16. *Ex vivo* examination of samples:
    - Arachidonic acid metabolism (platelets & aorta)
    - RNA isolation (platelets) and RT-qPCR
