# Supplementary material for: Effects of sub-chronic, in vivo administration of sigma-1 receptor ligands on platelet and aortic arachidonate cascade in streptozotocin-induced diabetic rats
Source: PLoS One. 2022 Nov 17;17(11):e0265854. doi: 10.1371/journal.pone.0265854 (PMC9671357; doi:10.1371/journal.pone.0265854)
Supplement: S3 Appendix — (PDF) [file pone.0265854.s003.pdf]

ADDITIONAL INFORMATION ON SAMPLING PROTOCOLS

1. Time point: 20 hours after the last i.p. administration of S1R ligand (PRE-084, *(S)*-L1 or NE-100)

2. Anaesthesia (30 mg/kg body weight Euthasol®/sodium pentobarbital i.p.)

3. Opening of the abdomen and exploration of the abdominal (dorsal) aorta

4. Drainage of 3, 2 and 7 ml (12 ml in total / rat) of blood from the abdominal aorta and dilution with double/double phosphate buffer (pH 7.4) containing EDTA (5.8 mM) and glucose (5.55 mM)

5. Euthanasia (100 mg/kg body weight Euthasol®/ sodium pentobarbital i.p.)

6. Isolation and resection of the aorta

Use of blood samples per group of animals (9 rats / group)

3 ml of blood

- plasma was separated and used to determine
- concentrations of S1R ligands, which was 1 sample per rat (i.e. 9 rats = 9 samples)
- serum metabolite levels (total cholesterol, ALT, BUN) from plasma remaining after serum ligand determination after pooling (after a simple randomization, 3 samples per group were generated from plasma samples of 9 rats, 3 rats each)

2 ml of blood

- platelet isolation and arachidonic acid metabolism
- 1-C^{14}-arachidonic acid substrate and ELISA (i.e. 9 rats = 9 samples)

7 ml of blood

- platelet isolation and RT-qPCR of *Sigmar1, Ptgs1, Ptgs2* genes

After simple randomization blood samples (~7 ml in each) from 9 rats in each treatment group were randomly allocated to 3 groups with 3 samples in each. The blood was pooled resulting in 3 biological samples/treatment group. Platelets were isolated separately from the 3 pooled blood samples (each representing 3 animals) and used for RNA isolation. The 3 biological samples from each treatment group represent a total of 9 rats.

Use of aorta per group (9 rats/group)

- parallel analysis of arachidonic acid metabolism with 1-C^{14}-arachidonic acid and ELISA (i.e. 9 rats = 9 samples)
