# Supplementary figures and images for: Effects of sub-chronic, in vivo administration of sigma-1 receptor ligands on platelet and aortic arachidonate cascade in streptozotocin-induced diabetic rats
Source: PLoS One. 2022 Nov 17;17(11):e0265854. doi: 10.1371/journal.pone.0265854 (PMC9671357; doi:10.1371/journal.pone.0265854)

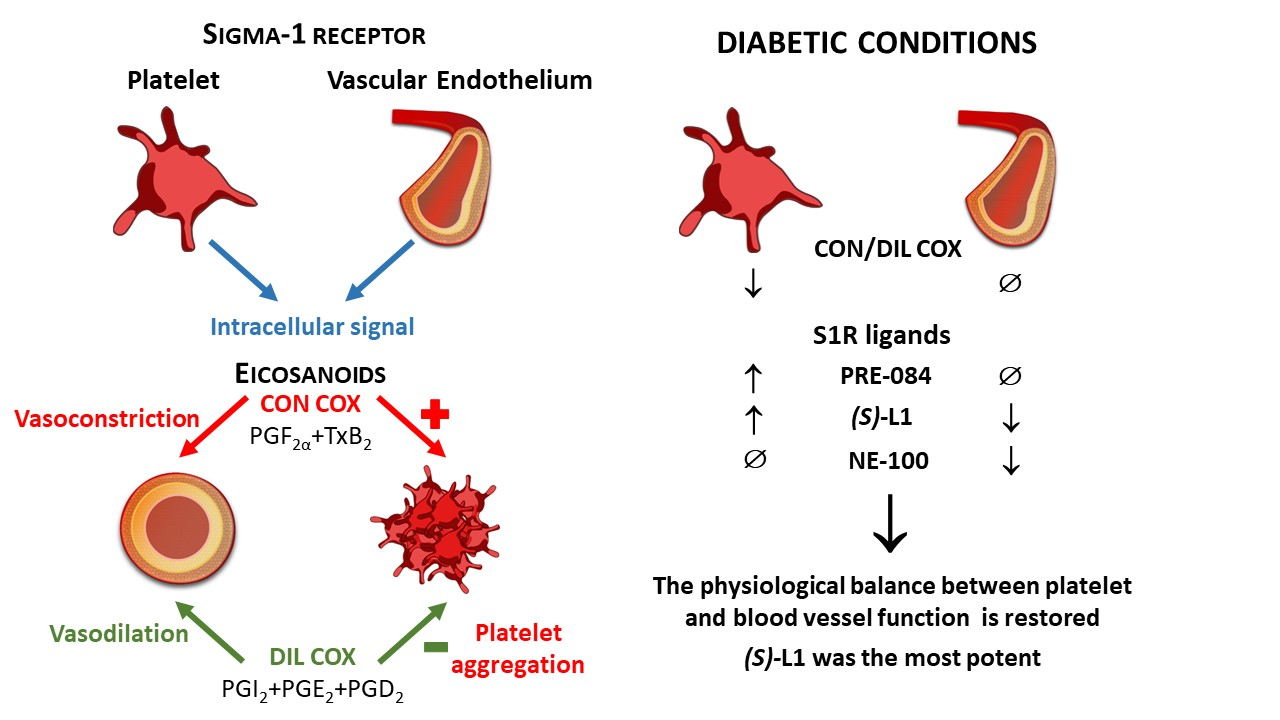

Supplement: S1 Graphical abstract — (TIF) [file pone.0265854.s004.tif]
